# Genome editing of susceptibility gene *StDND2* enhances *Phytophthora* resistance in *Solanum tuberosum*

**DOI:** 10.3389/fpls.2026.1807632

**Published:** 2026-06-05

**Authors:** Arsh Bibi, Irshad Ullah, Shaheen Aftab, Imran Amin, Bushra Bibi, Roger Schneiter

**Affiliations:** 1 Agricultural Biotechnology Division, National Institute for Biotechnology and Genetic Engineering (NIBGE), Constituent College Pakistan Institute of Engineering and Applied Sciences (PIEAS), Faisalabad, Pakistan; 2 Department of Biological Sciences, National University of Medical Sciences (NUMS), Rawalpindi, Pakistan; 3 National Institute of Genomics and Advance Biotechnology (NIGAB), National Agriculture Research Centre (NARC), Islamabad, Pakistan; 4 Department of Biology, University of Fribourg, Fribourg, Switzerland

**Keywords:** calcium signaling, DND2 gene, genome editing, oomycete resistance, plant defense, potato breeding, susceptibility gene, transgenic crops

## Abstract

Potato (*Solanum tuberosum L.)* cultivation is severely constrained by multiple pathogens, among which late blight caused by the oomycete *P. infestans* remains the most destructive disease. Developing pathogen-resistant cultivars can enhance productivity and reduce fungicide use in potato, where R-gene-based resistance is often overcome by evolving pathogen populations. Targeting susceptibility (S) genes represents a promising alternative strategy for improving disease resistance. Here, we employed CRISPR/Cas9 genome editing to introduce a targeted missense single-nucleotide polymorphism in the susceptibility gene *StDND2*, a putative susceptibility-associated cyclic nucleotide-gated channel family gene showing sequence similarity to *Arabidopsis thaliana DND2*, to evaluate its association with late blight resistance. The *StDND2* sequence was retrieved from the Spud DB database, and guide RNAs targeting the first exon were designed, Cas-OFFinder was used for off-target assessment. *Agrobacterium tumefaciens*-mediated transformation generated kanamycin-resistant edited lines, which were confirmed by PCR amplification of the Cas9 transgene. Resistance was evaluated using detached leaf assays following inoculation with *Phytophthora infestans*, and lesion areas were measured at 7 days post-inoculation. Edited lines showed significantly reduced lesion sizes (~74% lower infection rates) compared with empty-vector control plants, without obvious visible developmental abnormalities under the tested growth conditions after visual inspection. These findings provide preliminary evidence that CRISPR/Cas9-mediated editing of *StDND2* is associated with reduced late blight severity in potato under controlled experimental conditions and support further evaluation in advanced generations and field environments

## Introduction

Potato (*Solanum tuberosum* L.) is identified as the world’s fourth most significant staple crop after rice, wheat, and maize, based on its contribution to daily human calorie intake, accounting for approximately 1.7% of global consumption according to recent analyses up to 2025 (Aguilera-Galvez et al., 2018; Burgos et al., 2020; Barbaś et al., 2023). This ranking highlights the vital role of potato in food security, particularly in underdeveloped and developing regions, where it serves as an accessible source of carbohydrates, vitamins, and minerals.

Despite its adaptability and high productivity across diverse agroecological zones, potato cultivation is severely constrained by multiple pathogens, among which late blight caused by the oomycete *P. infestans* remains the most destructive disease, historically exemplified by the Irish Potato Famine and continuing to threaten global production (Fry et al., 2015; Ivanov et al., 2021). Despite advances in crop protection and breeding, late blight remains a continuing constraint due to the rapid evolutionary potential of *P. infestans* and its ability to overcome host resistance. Under favorable environmental conditions, epidemics can develop rapidly and cause severe to complete crop losses, figuring the need for durable, genetics-based resistance strategies (Coomber et al., 2024). *P. infestans* exhibits a hemibiotrophic lifestyle, initiating infection through a biotrophic phase that allows colonization of living host tissue, followed by a necrotrophic phase characterized by extensive tissue damage and sporulation (Kamoun and Smart, 2005; Lamichhane et al., 2024). This infection strategy enables the pathogen to manipulate host cellular processes while actively suppressing immune responses during early stages of infection. Such suppression is frequently facilitated through host regulatory genes that negatively modulate immune signaling pathways, creating susceptibility windows that favor pathogen establishment (Thakur et al., 2019).

Control of late blight relies largely on chemical fungicides such as mancozeb and chlorothalonil, which sustain substantial economic and environmental costs and promote the emergence of resistant pathogen populations. Therefore, late blight causes global annual losses exceeding $6.7 billion, with yield reductions of 50–70% reported in Pakistan and complete crop failure under epidemic conditions. These experiences emphasize the limitations of chemical control and reinforce the need for host-mediated resistance approaches that reduce reliance on fungicides (Tsedaley, 2014; Hari et al., 2025).

Plant immune responses against pathogens are coordinated through a multilayered defense system involving pattern-triggered immunity (PTI) and effector-triggered immunity (ETI) (Jones and Dangl, 2006; Thomma et al., 2011; Miller et al., 2017). PTI is activated upon recognition of pathogen-associated molecular patterns by pattern recognition receptors, whereas ETI is induced through recognition of pathogen effectors by host resistance (*R*) proteins. Although *R*-gene-mediated resistance has been significantly exploited in potato breeding, its durability is often compromised due to the rapid evolution of virulent *P. infestans* strains (Sun et al., 2016; Leesutthiphonchai et al., 2018; Yu et al., 2024). Traditional resistance breeding has relied heavily on introgression of *R*-genes from wild potato species such as *Solanum demissum*, resulting in the deployment of at least eleven major *R*-genes (*R1–R11*) in cultivated potato (Reena Sharma et al., 2013). However, the efficiency of these genes is frequently short-lived, as pathogen populations adapt quickly to overcome single-gene resistance. Additionally, potato’s tetraploid genome, vegetative propagation, and linkage drag significantly complicate conventional breeding efforts. These challenges limit the long-term effectiveness of R-gene-based strategies and highlight the need for alternative approaches for improving disease resistance in potatoes. An emerging alternative strategy focuses on the manipulation of host susceptibility (*S*) genes that are utilized by pathogens to facilitate infection (Pessina, 2016). Loss-of-function mutations in *S*-genes can confer recessive but often more durable resistance by removing host factors required for pathogen success. *S*-genes involved in immune suppression, nutrient transport, or host recognition are particularly attractive targets for engineering disease resistance. Unlike *R*-genes, which exert strong selective pressure on pathogen populations, *S*-gene-based resistance is thought to impose lower evolutionary pressure, thereby enhancing durability (Thomazella et al., 2021; Huang et al., 2023).

Among negative regulators of plant immunity*, the Defense No Death 2 (DND2)* gene represents a well-characterized negative regulator of plant immunity.*DND2* encodes a cyclic nucleotide-gated calcium channel (CNGC) involved in Ca²^+^-dependent signaling pathways that modulate immune responses (Zhang et al., 2014). In *A. thaliana*, loss of *DND2* function results in constitutive defense activation without triggering hypersensitive cell death, highlighting its role in fine-tuning immune signaling. *StDND2*, a putative potato homolog of *Arabidopsis AtCNGC4* (Soltu.DM.12G022320.1), is homologous to *Arabidopsis AtCNGC4* and is predicted to play a similar regulatory role in calcium-mediated defense signaling (Jurkowski et al., 2004; Genger et al., 2008; Roy et al., 2020). *StDND2* belongs to the cyclic nucleotide-gated channel (CNGC) gene family, which comprises calcium-permeable ion channels conserved across plant species and functionally associated with regulation of immune signaling and stress responses.Despite the well-established function of *DND2* in *Arabidopsis*, its specific contribution to late blight susceptibility in potato has not been comprehensively functionally validated. Given the complexity of calcium signaling networks and species-specific immune regulation, functional extrapolation from model plants to potato cannot be assumed (Sun et al., 2022; Bánfalvi et al., 2024; Sun et al., 2024). Moreover, calcium-permeable channels often affect multiple signaling pathways so the editing of CNGC genes may cause certain trade-offs such as between growth-defense balance or changes in plant abiotic stress responses, so it needs careful evaluation in potato (Chin et al., 2013).

Recent advances in genome editing technologies, particularly the CRISPR/Cas9 system, have enabled precise and efficient modification of plant genomes for disease resistance (Puchta et al., 1993; Kieu et al., 2021; Tiwari et al., 2022). CRISPR/Cas9-mediated editing of S-genes has been successfully applied in potato to enhance resistance against *P. infestans* by targeting genes such as *StDND1*, *StDMR6*, *StBIK1*, *StCeSA3*, and *StCHL1* (Kieu et al., 2021; Bi et al., 2024; Karlsson et al., 2024; Norouzi et al., 2024). These studies demonstrate the feasibility of S-gene editing for improving disease resistance while minimizing negative effects on plant growth. However, susceptibility genes associated with calcium channel mediated immune regulation remain comparatively underexplored in potato, despite their central role in signal transduction during pathogen attack (Razzaq et al., 2022; Bi et al., 2023). Previous studies using RNA interference (RNAi) have reported that transcriptional silencing of *StDND2* reduces susceptibility to *P. infestans* in potato, supporting its role as a negative regulator of plant immunity. These findings provide initial functional evidence for *StDND2* as a susceptibility-associated gene in potato and highlight its potential as a target for genetic improvement (Jahan et al., 2015; Sun et al., 2016, 2022).

In the present study, we employed CRISPR/Cas9-mediated genome editing to generate targeted mutations in *StDND2* of *S. tuberosum*. In contrast to RNAi-based approaches that result in partial and variable gene silencing, CRISPR/Cas9 enables targeted modification of the coding sequence, allowing targeted alteration of gene function through sequence modification at the target locus. By functionally validating *StDND2* as a susceptibility gene in potato, this work aims to expand the repertoire of candidate S-genes for improving late blight resistance. We further assess whether targeted editing of *StDND2* is associated with reduced late blight severity without obvious visible developmental abnormalities under the tested conditions, thereby suggesting its potential for future evaluation in breeding programs, pending validation in advanced generations and field conditions.

## Materials and methods

### Plant material

The tetraploid potato (*Solanum tuberosum* L.) cultivar ‘Kuroda’ was used for genetic transformation. *In vitro* plantlets were maintained on Murashige and Skoog (MS) basal medium supplemented with 30 g/L sucrose, 100 mg/L myo-inositol, 1 mL/L MSVI vitamin mix, 1 mL/L JHMS vitamin mix, and 8 g/L agar (pH 5.6–5.8). Internodal stem segments from six-week-old plants served as explants.

### Identification of target gene and gRNA design

*StDND2* (PGSC0003DMG400025027) from Ensembl Plants, or (Soltu.DM.12G022320.1) was selected on the basis of sequence homology to *Arabidopsis thaliana DND2/CNGC4* (*AtCNGC4* AT5G54250) (Jurkowski et al., 2004). Reciprocal BLAST was performed by querying the Arabidopsis thaliana *DND2* protein sequence against the *Solanum tuberosum* proteome using BLASTp, followed by re-querying the top potato hit against the Arabidopsis proteome to confirm orthology based on recovery of *DND2* as the best reciprocal match; additionally, the nucleotide sequence of the top *Solanum tuberosum* hit was subjected to reverse BLASTn against the genomes of *Solanum lycopersicum* and *Capsicum annuum* to further validate sequence conservation across related species (**Supplementary Tables S1, S2**). Further, to confirm the putative ortholog of Arabidopsis *DND2/CNGC2* in potato, the Arabidopsis protein sequence was used as a query for protein BLAST searches against plant protein databases in Ensembl Plants (https://plants.ensembl.org/). Homologous protein sequences from representative plant species were aligned, and a Maximum Likelihood phylogenetic tree was constructed using MEGA11. Guide RNAs targeting the first exon were designed using Cas-OFFinder; potential off-target sites were evaluated against the Ensembl Plants potato genome. Two complementary DNA oligonucleotides incorporating BbsI-compatible overhangs were synthesized (**Table 1**).

**Table 1:** Primer sequences used to amplify U6 promoter and gRNA scaffold.

Gene name	Forward DNA Oligo (5’- 3’)	Reverse DNA oligo (5’- 3’)
*DND2*	ATTGTCACTAAATCGATATTTC	AAACGAAATATCGATTTAGTGA
	ATTGAAGCGACAGCAGTCCAAC	AAACTTCGCTGTCGTCAGGTTG
p-c-*HindIII*	TCCAAAGCTTCCTAGGCTTTTTTTCTTC	CCAGAAGCTTCTAGGTAATGCCAACTTT

Overhangs (indicated in red) containing *BbsI* restriction sites were added to the 5′ ends of each oligonucleotide to enable directional ligation of the annealed gRNA into the sgRNA expression cassette within PK2-GW7-Cas9.

### CRISPR/Cas9 vector construction and cloning

The sgRNA cassette was assembled into the Ptz-p-chimera vector (Hameed et al., 2017), containing the *A. thaliana* U6–26 promoter and sgRNA scaffold, and subsequently ligated into the binary vector PK2-GW7-Cas9 (Ali et al., 2015) via *HindIII* digestion and T4 DNA ligation (detailed cloning workflow in **Supplementary Figure 1**). The assembled CRISPR/Cas9 constructs were first introduced into *Escherichia coli* for plasmid amplification and verification prior to transformation into *Agrobacterium tumefaciens*. The final construct was verified by PCR and restriction digestion (**Supplementary Figure 2**). Primer sequences are listed in **Table 1**.

### Transformation of *Agrobacterium tumefaciens*

The confirmed CRISPR/Cas9 binary constructs carrying the *StDND2* sgRNA cassette were introduced into *Agrobacterium tumefaciens* strain LBA4404 by electroporation using an Electro-Cell Manipulator 600 (BTX Harvard Apparatus, USA). Electrocompetent cells (70 µL) were mixed with 2 µL plasmid DNA in pre-chilled 1 mm gap cuvettes and pulsed at 1.44 kV (≈6 ms). Immediately after pulsing, 1 mL C-medium (Thermo Fisher Scientific, USA) was added, and the suspension was incubated at 28 °C for 2 h with shaking (180 rpm) for recovery.

Transformed cells were plated on selective agar containing rifampicin (50 µg/mL) and the appropriate antibiotic for the binary vector and incubated at 28 °C for 48 h. Ten bacterial colonies per construct were screened by colony PCR using primers specifically for the PK2-GW7-Cas9 vector and the *StDND2* gRNA cassette. Positive transformants were grown overnight (24–48 h) at 28 °C in liquid medium and adjusted to an optical density (OD_600_) of approximately 0.5–0.8 prior to infection. Explants were incubated with the bacterial suspension and co-cultivated on co-cultivation medium for 2–3 days under dark conditions before transfer to selection medium. Empty-vector control plants were generated by transformation with the CRISPR/Cas9 vector lacking the gRNA cassette, to control for potential effects of the transformation process and vector backbone.Transformation was performed following a previously described protocol with modifications (Zhang et al., 2020; Ruzyati et al., 2022).

### Efficient generation of transgenic potato lines

Explants were co-cultured for 48 h on 3C5ZR medium (**Table 2**), then transferred to callus induction medium (CIM) containing kanamycin (50 mg/L) and timentin (300 mg/L). Regenerated shoots were rooted on MS medium with kanamycin. Media compositions are detailed in **Table 2**.

**Table 2:** Composition of plant tissue culture media used for *Agrobacterium*-mediated transformation of *Solanum tuberosum*.

3C5ZR medium 1L		Callus induction medium (CIM) 1L	
Murashige and Skoog Basal Salt Mixture	4.3 g	Murashige and Skoog Basal Salt Mixture	4.3 g
Sucrose	30g	Sucrose	30 g
Myo-inositol	100 mg	Myo- inositol	100 mg
Vitamins 3R (stock)	1000 µL	Vitamins MSV1 (stock)	1000 µL
Zeatin riboside (1.0 mg/mL)	3000 µL	vitamins JHMS (stock)	1000 µL
Kanamycin (50.0 mg/mL)	500 µL	NAA (1.0 mg/mL)	2000 µL
IAA (1.0 mg/mL)	500 µL	BAP (1.0 mg/mL)	1000 µL
Timentin	3000 µL		
Agar	4 g		4 g
pH	5.8		5.8

### PCR-based validation of Cas9 and selection marker integration

Genomic DNA was extracted and screened by PCR for Cas9 and kanR integration. Targeted amplicon sequencing of the *StDND2* locus was performed using the primers in **Table 3**. Library preparation, Illumina sequencing, quality control (FastQC, fastp, MultiQC), alignment (BWA), and variant calling (GATK HaplotypeCaller) followed standard pipelines (full details in **Supplementary Material**).

**Table 3:** Gene specific primers for amplicon sequencing.

Genes	Forward primer	Reverse primer
*DND2*	TAAACTTGATTTGACGAGTTG	CTCCGACTAGATATCACTAG

### Detached leaf bioassay

Resistance to *Phytophthora infestans* isolate US-23 (University of Wisconsin–Madison) was evaluated using the detached-leaf assay of Karki and Halterman (2021). The isolate was routinely maintained on Rye A agar medium and stored at 18 °C under dark conditions. For long-term preservation, cultures were periodically subcultured to maintain viability and pathogenicity. Each treatment included 4–6 leaflets per plant, and the experiment was repeated independently four times. Data from independent experimental repeats is provided in the **Supplementary Material**.

### Statistical analysis

**Figure 1:**
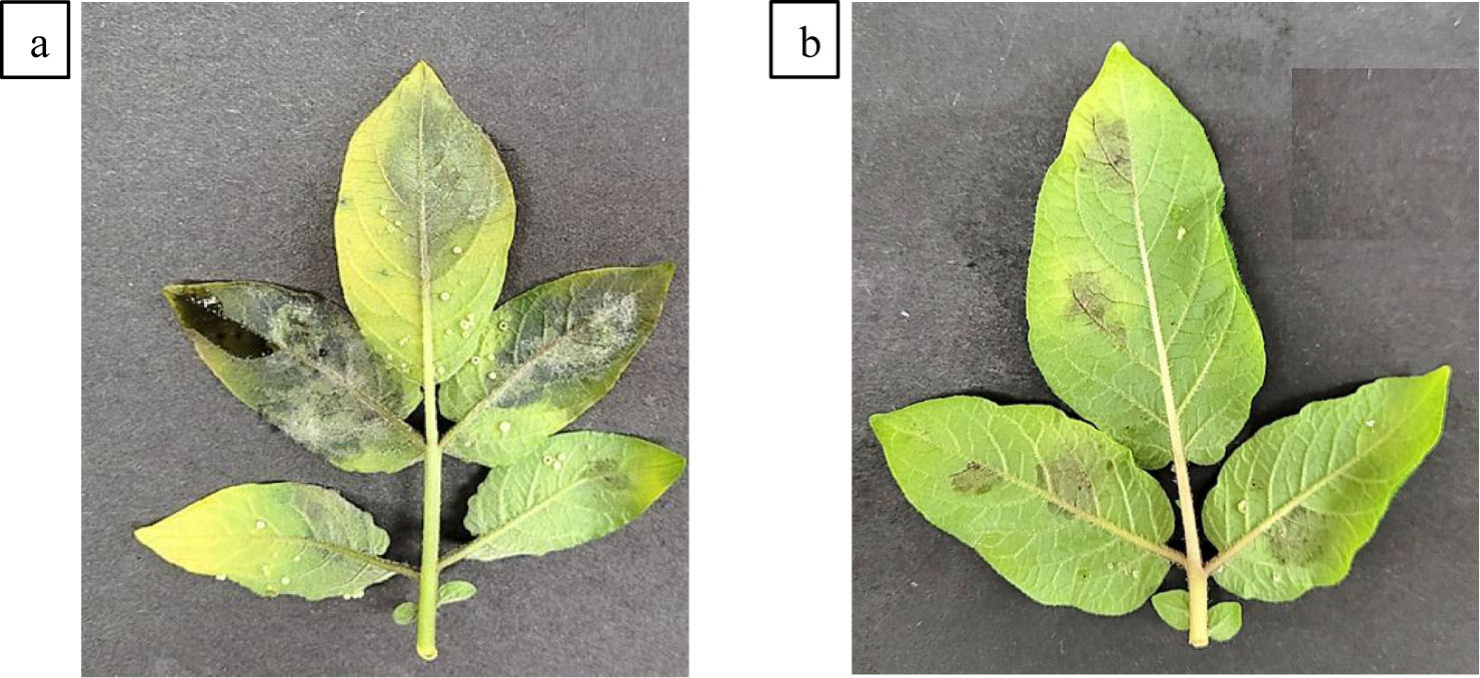
**(A)** Detached leaf assay showing disease symptoms caused by *Phytophthora infestans* isolate US-23 at 7 days post-inoculation (dpi). Representative leaflets from empty-vector control plants (left) and *StDND2*-edited plants are shown. **(B)** Edited plants exhibit visibly reduced lesion development compared with control plants under identical inoculation conditions. Leaflets were inoculated on the abaxial surface with sporangial suspensions and incubated under controlled environmental conditions (~21 °C, high humidity). Images represent independent biological replicates. Sporangial suspensions were applied as defined droplets to each leaflet to ensure uniform infection, and disease progression was assessed under identical experimental conditions across all samples.

All experiments were conducted using a minimum of three independent biological replicates, unless otherwise stated. Quantitative data obtained from lesion area measurements in detached leaf assays were used for statistical evaluation. Data are presented as mean ± standard deviation (SD), unless otherwise indicated. For disease severity analysis, four independent biological replicates (n = 4) were used, consistent with the data presented in **Figure 1**. Statistical comparisons between genome-edited lines and empty-vector controls were performed using appropriate statistical tests selected based on data distribution and experimental design. For comparisons involving multiple edited lines and controls, statistical significance was evaluated using one-way ANOVA followed by Dunnett’s multiple-comparison test using the empty-vector control (PK/A) as the reference group. For pairwise comparisons between edited and control groups in detached leaf assays, an unpaired two-tailed Student’s t-test was applied. Statistical significance was defined at p < 0.05, and values of p < 0.01 were considered highly significant. The specific statistical tests applied are indicated in the corresponding figure legends. The value of n represents independent biological replicates (independent experimental repeats), unless otherwise stated. For tuber number assessment, three independent biological replicates were evaluated for each edited line and empty-vector control plant under controlled growth conditions.

### Protein structure prediction

Wild-type and edited *StDND2* amino-acid sequences were submitted to the TrRosetta server. Models were superimposed in UCSF Chimera v1.8 to assess conformational differences (detailed results presented in Results section).

## Results

### CRISPR/Cas9 construct successfully assembled and validated

**Figure 2:**
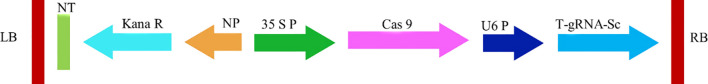
Schematic representation of the CRISPR/Cas9 binary vector used for targeted mutagenesis of *StDND2* in *Solanum tuberosum*. The T-DNA region contains a codon-optimized *Cas9* endonuclease driven by the Cauliflower mosaic virus 35S promoter (35S P). The single guide RNA (sgRNA) expression cassette comprises the *Arabidopsis thaliana* U6–26 promoter (U6 P) driving the gRNA scaffold fused to a 22-bp target sequence specific to *StDND2*. The vector also carries a kanamycin resistance gene (Kana R) under the control of the nopaline synthase promoter (NP) and terminator. Left border (LB) and right border (RB) sequences define the T-DNA region used for *Agrobacterium-*mediated plant transformation. The sgRNA target sequence was designed to specifically target the first exon of *StDND2* adjacent to a protospacer adjacent motif (PAM) site.

The ortholog of *AtDND2* in potato was confirmed by reciprocal BLAST and phylogenetic tree construction. The candidate potato protein clustered closely with Solanaceae homologs within the same conserved CNGC clade as Arabidopsis *DND2/CNGC2*, supported by high bootstrap values, indicated that the identified potato gene *StDND2* is the likely ortholog of *AtDND2/CNGC2* (**Supplementary Figure 5**). After gene orthology confirmation, to generate a CRISPR/Cas9 construct targeting the *StDND2* susceptibility gene, two guide RNAs (gRNAs) were designed to target conserved regions within the first exon of *StDND2* (Soltu.DM.12G022320.1), adjacent to protospacer adjacent motif (PAM) sites, following established CRISPR/Cas9 design principles for plant genome editing (Jaganathan et al., 2018; Kieu et al., 2021). In silico off-target analysis using Cas-OFFinder in combination with the Ensembl Plants genome database did not identify potential off-target sites with up to three mismatches, supporting the specificity of the selected gRNAs (Bae et al., 2014). The sgRNA expression cassette, comprising the *A. thaliana* U6–26 promoter and gRNA scaffold, was assembled using a stepwise cloning strategy (**Supplementary Figure 1**), consistent with previously reported plant CRISPR vector systems (Schumacher et al., 2017; Li et al., 2021). A *BbsI* restriction site located between the U6 promoter and sgRNA scaffold enabled directional insertion of annealed gRNA oligonucleotides (**Supplementary Figures 1A, B**). The assembled U6–sgRNA cassette was first ligated into the TA cloning vector Ptz57rt to generate the intermediate Ptz-p-chimera construct (**Supplementary Figures 1C, D**), facilitating subsequent transfer into the CRISPR/Cas9 binary vector. Final assembly of the CRISPR/Cas9 expression construct was achieved by ligation of the U6–sgRNA cassette into the PK2-GW7-Cas9 binary vector, which contains a codon-optimized Cas9 gene driven by the CaMV 35S promoter and a kanamycin resistance marker for plant selection (**Figure 2**; **Supplementary Figure 1E**). Successful construct assembly was verified by PCR amplification of the U6–sgRNA cassette, yielding the expected 545 bp product, and by *HindIII* restriction digestion, which confirmed loss of the diagnostic restriction fragment following cassette insertion (**Supplementary Figure 2A**).

### Generation and selection of transgenic potato plants

**Figure 3:**
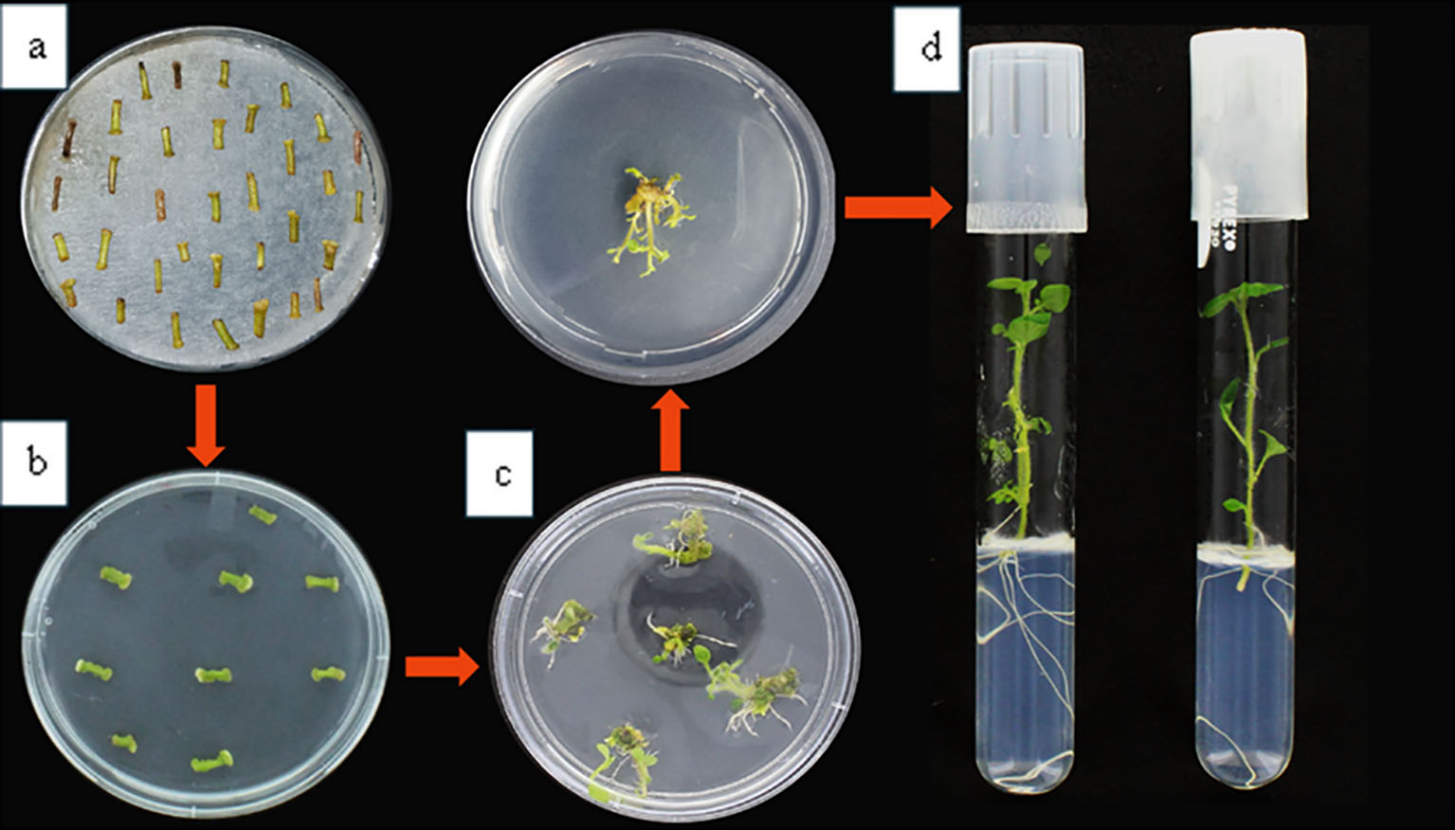
Stages of *Agrobacterium* mediated transformation and regeneration of potato (*Solanum tuberosum* cv. Kuroda’). **(A)** Internodal stem explants excised from *in vitro*-grown plants and subjected to *A. tumefaciens* infection. (Explants were co-cultivated with *Agrobacterium* carrying the CRISPR/Cas9 construct under controlled conditions to facilitate T-DNA transfer). **(B)** Callus induction from transformed explants on callus induction medium (CIM). (Explants were cultured under selective conditions to promote callus formation from transformed tissues). **(C)** Shoot regeneration from calli on 3C5ZR regeneration medium. (Regeneration medium was supplemented with plant growth regulators to induce shoot formation). **(D)** Regenerated shoots developing roots on MS0 medium supplemented with 50 mg/L kanamycin for selection of putative transgenic plants. (Kanamycin selection was used to eliminate non-transformed tissues and confirm stable integration of the construct).

**Figure 4:**
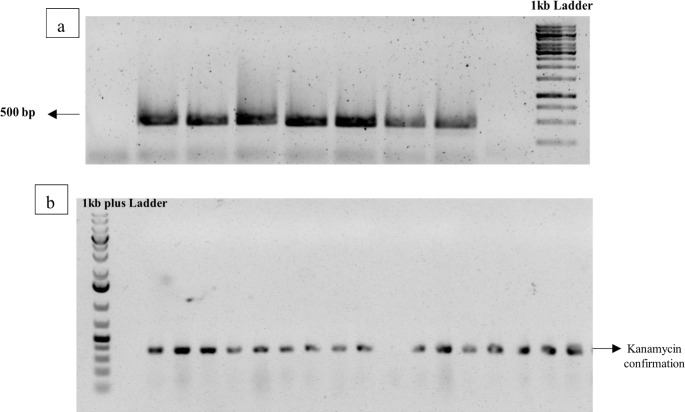
PCR-based confirmation of transgenic potato plants. **(A)** Agarose gel electrophoresis showing amplification of the Cas9 transgene (~500 bp) in putative transgenic lines (lanes 2–9); lane 1 corresponds to the non-transformed wild-type control. A 1 kb DNA ladder is shown on the right. PCR amplification was performed using gene-specific primers targeting the Cas9 coding region, and the presence of the expected band indicates successful integration of the transgene. (**Supplementary Figure 2**). **(B)** Agarose gel electrophoresis showing amplification of the kanamycin resistance gene (~330 bp) in putative transgenic lines (lanes 2–18); lane 1 corresponds to the non-transformed wild-type control. A 1 kb Plus DNA ladder is shown on the left. Amplification of the selectable marker gene further confirms the presence of the T-DNA construct in regenerated lines.

The validated CRISPR/Cas9 construct targeting *StDND2* was introduced into *A. tumefaciens* strain LBA4404 and used for genetic transformation of internodal explants derived from *in vitro*-grown potato (*S. tuberosum* cv. Kuroda’) plants. Internode segments (30–50 explants per batch) were subjected to *Agrobacterium-*mediated transformation and cultured through sequential stages of callus induction, shoot regeneration, and selection (**Figure 3**). From approximately 5,000 infected explants, 90 explants produced multiple shoots under selection conditions, corresponding to an overall transformation efficiency of approximately 9%. Regeneration proceeded through callus formation on callus induction medium, followed by shoot induction on 3C5ZR medium and subsequent rooting on MS0 medium supplemented with 50 mg/L kanamycin (**Figures 3A, B**; **Supplementary Table 2**). Explants exhibiting bleaching, failure to root, or abnormal aerial root formation were excluded from further analysis. A total of 26 putative transgenic lines with successful shoot regeneration and root establishment were recovered and advanced for molecular validation. Each well-rooted plantlet was maintained separately during downstream analyses. PCR analysis confirmed amplification of a 500 bp fragment corresponding to the *Cas9* gene in transgenic lines, while no amplification was detected in non-transformed control plants (**Figure 4A**). In parallel, PCR amplification of the kanamycin resistance gene yielded the expected 330 bp product in the same set of transgenic lines (**Figure 4B**). Only positive lines for both selectable markers were retained for subsequent analyses.

### Mutation confirmation by targeted sequencing

**Figure 5:**
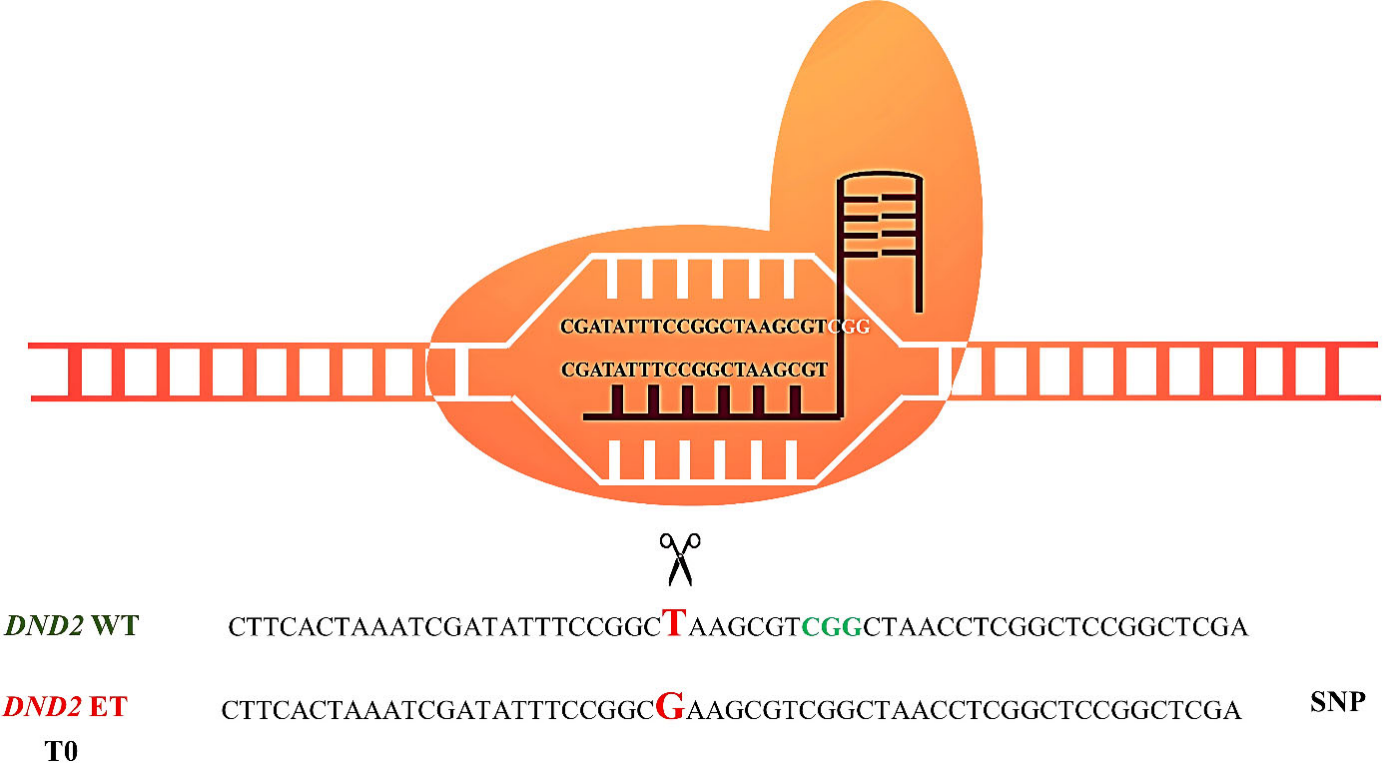
Schematic representation of CRISPR/Cas9-mediated editing of *StDND2*. Sequence comparison between the wild-type (WT) and edited *StDND2* alleles showing the T→G nucleotide substitution at the predicted Cas9 cleavage site within the target region. The edited nucleotide is highlighted in red. The guide RNA target sequence and protospacer adjacent motif (PAM) are indicated schematically. Sequences were derived from targeted amplicon sequencing of the *StDND2* locus.

**Figure 6:**
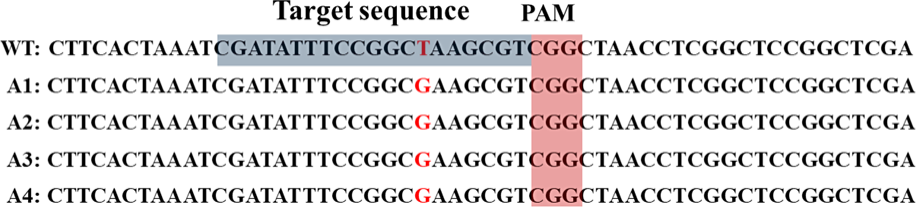
Targeted next-generation sequencing confirms CRISPR/Cas9-mediated editing of *StDND2*.Alignment of Illumina sequencing reads to the *StDND2* reference sequence showing a consistent T→G nucleotide substitution at the predicted CRISPR/Cas9 cleavage site within the first exon. The wild-type (WT) sequence retains the unedited nucleotide, all analyzed allelic sequence variants in the edited line carry the same substitution, consistent with editing across the analyzed alleles. Read coverage and base substitutions were visualized using Integrative Genomics Viewer (IGV). The edited allele was consistently detected in both T0 and T1 generations under the conditions tested.

To characterize CRISPR/Cas9-induced mutations at the *StDND2* target locus, targeted next-generation sequencing (NGS) was performed on genomic DNA isolated from edited T0 plants and their corresponding T1 progeny. Sequence analysis revealed a consistent T→G nucleotide substitution at the predicted Cas9 cleavage site within the first exon of *StDND2*, and a consistent editing signal was observed across all allelic copies in the analyzed edited lines (**Figure 5**). In the edited line analyzed, the substitution was detected across all analyzed allelic sequence variants. This nucleotide substitution was consistently detected at the target site in both T0 and T1 generations under the conditions tested (**Figure 6**; **Supplementary Figure 3**). The T→G substitution resulted in a missense mutation leading to a leucine-to-glutamine amino acid change in the encoded protein. Sequence analysis further confirmed the presence of the edited nucleotide at the target locus, while the empty-vector control retained the unedited sequence (**Figure 6**). No additional insertions or deletions were detected at the target site. The consistent T→G substitution observed across the analyzed edited lines suggests that these plants may share a similar editing outcome; however, the independence of these editing events could not be conclusively determined based on the available data.

### Phenotypic assessment and tuber yield of edited plants

**Figure 7:**
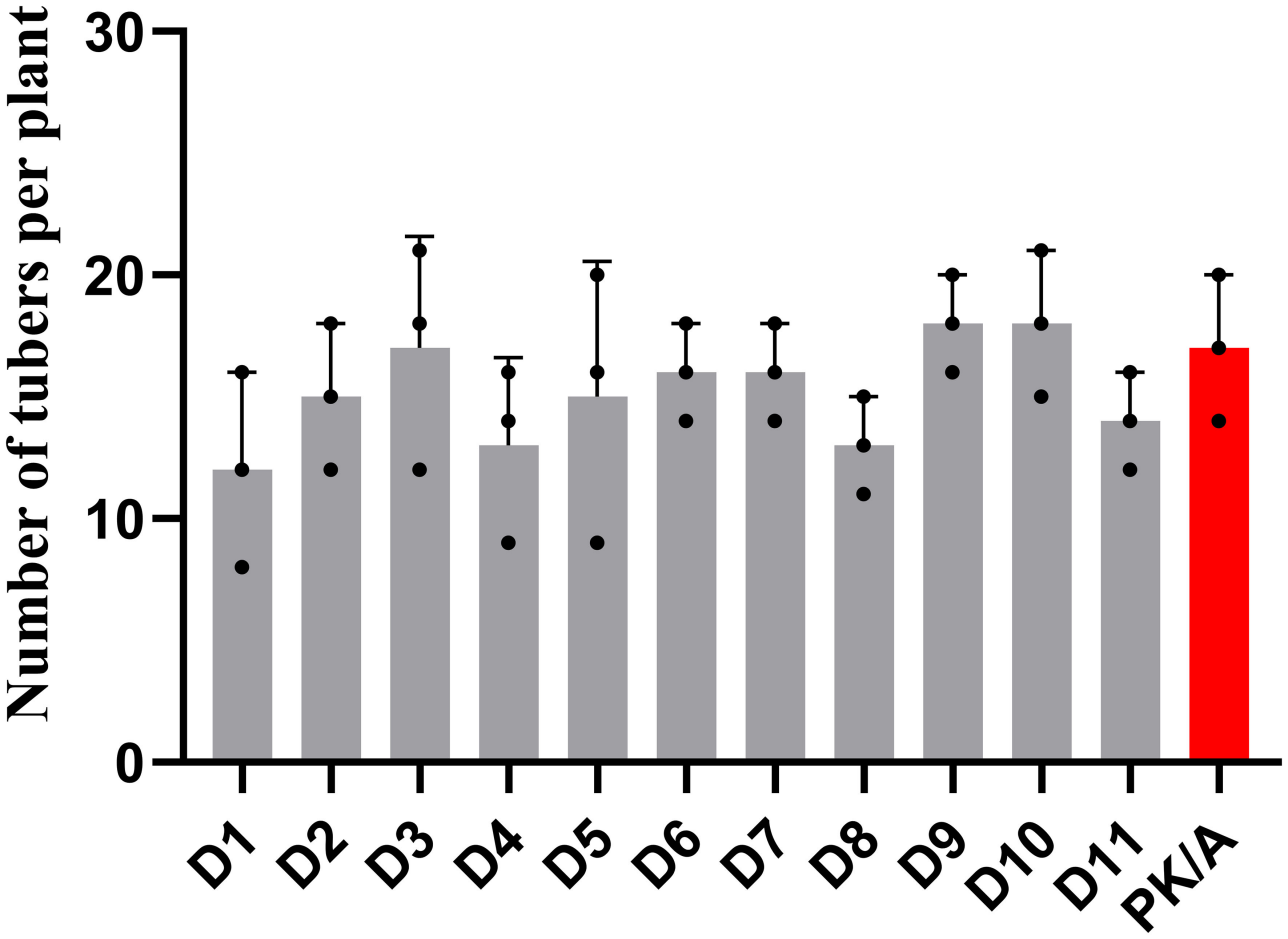
Tuber number assessment of analyzed *StDND2*-edited potato lines and empty-vector control plants under controlled growth conditions. Individual biological replicate values are shown (n = 3 independent biological replicates per line). Bars represent mean ± SD. Statistical analysis was performed using one-way ANOVA followed by Dunnett’s multiple-comparison test using the empty-vector control (PK/A) as the reference group. No significant differences were detected under the tested conditions (p > 0.05).

To evaluate potential pleiotropic effects associated with *StDND2* editing, edited plants were evaluated by visual inspection for morphology, and number of tuber production under standard growth conditions. No obvious qualitative differences in overall plant appearance were observed between edited and empty-vector control plants under the tested growth conditions. Quantitative analysis of tuber number revealed variation among the analyzed edited lines, ranging from 8–21 tubers per plant across biological replicates (**Figure 7**). Statistical analysis using one-way ANOVA followed by Dunnett’s multiple-comparison test showed no significant differences in tuber number between edited lines and the empty-vector control under the tested conditions (p > 0.05). These observations suggest that the edited lines did not exhibit obvious reductions in tuber numbers under the tested growth conditions (**Figure 7**).

### Detached leaf assay reveals reduced late blight susceptibility

**Figure 8:**
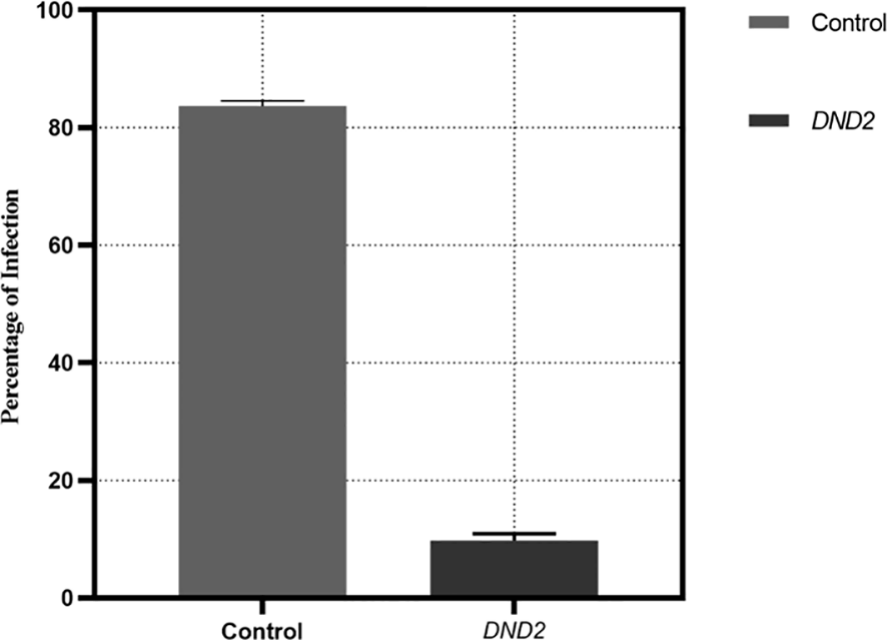
The bar graph shows the percentage of infected leaf areas in *StDND2*-edited plants compared with empty-vector control plants. Data are presented as mean ± SEM from four independent biological experiments (n = 4), with each replicate consisting of multiple leaflets (approximately four per treatment). Statistical significance was determined using an unpaired Student’s t-test (p < 0.01). Lesion areas were quantified using ImageJ software, and values represent average measurements from multiple leaflets per replicate.

To evaluate the impact of *StDND2* editing on resistance to late blight, detached leaf assays were performed using *P. infestans* isolate US-23 on leaves from 4–5-week-old plants. Disease development was evaluated at 7 days post-inoculation (dpi). Edited plants exhibited visibly reduced lesion development compared with empty-vector control plants (**Figures 1A, B**). Quantitative analysis of disease severity revealed that edited lines showed a mean infection level of approximately 10%, whereas control plants exhibited approximately 84% infection at 7 dpi (**Figure 8**). This represents an approximately 74% reduction in disease severity in edited plants. Statistical analysis confirmed that the difference between edited and control plants was significant (unpaired Student’s *t*-test, p < 0.01). These results indicate that targeted editing of *StDND2* is associated with reduced disease severity under detached leaf assay conditions.

### Structural modeling of the *StDND2* missense mutation

Amino acid sequences of both wild-type and CRISPR/Cas9-edited DND2 proteins (**Supplementary Materials**) were analyzed to predict and compare their three-dimensional structures, with the wild-type protein used as a reference. FASTA sequences corresponding to the edited and wild-type forms were submitted to the TrRosetta web server, which employs deep learning-based prediction of inter-residue distances and orientations to generate three-dimensional protein models. Predicted structures were visualized and superimposed using UCSF Chimera v1.8 to assess structural similarity and identify localized conformational differences, particularly in regions proximal to the mutation site. Sequence alignment identified a leucine-to-glutamine substitution within the conserved motif LSVGSGS, which was altered to QSVGSGS in the edited protein. Structural superimposition revealed strong conservation of the overall protein fold, with localized conformational changes restricted to the region surrounding the substituted residue. Replacement of the hydrophobic leucine with the polar glutamine is predicted to modify local hydrogen-bonding interactions and reduce hydrophobic packing, which may potentially influence local protein stability and conformational flexibility. These subtle structural perturbations are consistent with a partial modulation of DND2 function rather than complete structural disruption and may potentially influence local protein conformation and associated signaling properties. The comparative visualization of wild-type and edited protein structures highlighting these localized differences is shown in **Supplementary Figure 4**. These structure predictions are based on *in silico* modeling and are intended to provide qualitative insights into potential conformational effects; experimental structural validation would be required to confirm these predictions.

## Discussion

Late blight caused by *P. infestans* remains one of the most devastating diseases of potato worldwide, and the limited durability of resistance based on single dominant resistance (R) genes continues to challenge breeding efforts (Du and Vleeshouwers, 2017; Witek et al., 2021; Gopal, 2023; Wang et al., 2026). Recent advances in resistance engineering suggest that susceptibility gene modification and immune receptor optimization may contribute to improved disease resistance under specific experimental conditions. In this study, targeted CRISPR/Cas9-mediated editing of the potato susceptibility-associated gene *StDND2* was associated with a substantial reduction in late blight severity under detached leaf assay conditions, without obvious visible developmental abnormalities under the tested conditions. Edited plants carrying nucleotide substitutions within the first exon of *StDND2* exhibited reduced disease severity under controlled experimental conditions. These findings provide preliminary support for the potential utility of host susceptibility gene modification as a strategy for investigating disease resistance under control conditions (Bi et al., 2023; Karlsson et al., 2024). Previous studies using RNA interference (RNAi) have reported that transcriptional silencing of *StDND2* reduces susceptibility to *P. infestans* in potato, providing initial functional evidence for its role as a susceptibility-associated gene (Sun et al., 2022). In this context, the present study extends these findings by employing CRISPR/Cas9-mediated genome editing to generate a nucleotide substitution within the coding region of *StDND2*, allowing targeted modification of gene function compared with transcriptional knockdown approaches. The resistance-associated phenotype observed in *StDND2*-edited plants is consistent with the established role of *DND* family genes as negative regulators of plant immunity. In *A. thaliana*, *DND2* encodes a cyclic nucleotide-gated calcium channel implicated in immune signaling pathways, and loss of *DND2* function has been shown to activate defense responses without triggering hypersensitive cell death (Genger et al., 2008; Wang et al., 2022). The identification of *StDND2* in this study was based on sequence homology and annotation within the potato reference genome, supported by conserved features characteristic of cyclic nucleotide-gated channels (CNGCs). Calcium fluxes are among the earliest cellular responses following pathogen perception and are tightly regulated to balance defense activation with plant growth and development. Negative regulators such as *DND2* are therefore important for preventing excessive or mis-timed immune responses (Ma et al., 2010; Wang and Luan, 2024; Li et al., 2026). Recent studies have further demonstrated that calcium-dependent protein kinases function as important regulators linking immune receptor activation to downstream defense responses in potato *P. infestans* interactions (Jurkowski et al., 2004; Genger et al., 2008; Zhao et al., 2025). Together, these observations are consistent with the possibility that *StDND2* may play a comparable regulatory role in potato immunity.

**Figure 9:**
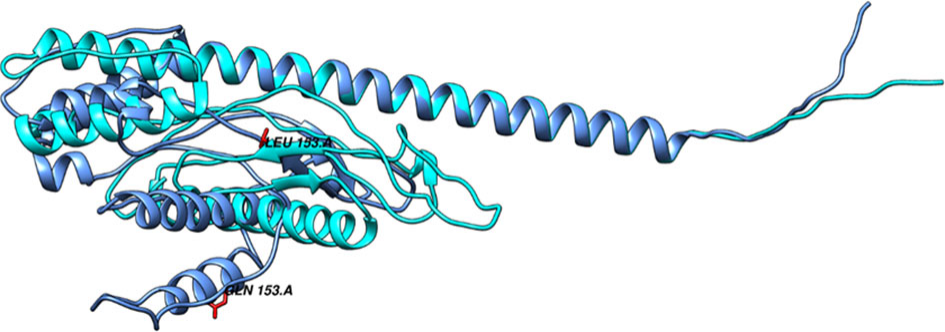
Structural comparison of wild-type and CRISPR/Cas9-edited StDND2 proteins. Predicted three-dimensional structures of the wild-type and edited StDND2 proteins were generated using TrRosetta and superimposed using UCSF Chimera v1.8. The overall protein fold is conserved between the two models, while localized conformational differences are observed in the region surrounding the edited residue. The mutation site corresponding to a leucine-to-glutamine substitution at position 153 (Leu153→Gln153) is highlighted, illustrating subtle structural alterations proximal to the conserved *LSVGSGS* motif. These localized changes are consistent with partial modulation of StDND2 protein function rather than complete structural disruption.

Although direct functional extrapolation between species must be interpreted cautiously, the resistance-associated phenotype observed in *StDND2*-edited potato plants is consistent with a conserved regulatory role for *DND2*-related genes across plant species. However, the classification of *StDND2* as a susceptibility-associated gene in potato is supported by functional evidence from genome editing and phenotypic analysis in this study rather than just sequence homology alone and should therefore be considered preliminary pending further mechanistic validation. Similarly, disruption of key metabolic regulators such as *SnRK1β1A* has been shown to enhance broad-spectrum disease resistance through modulation of immune signaling pathways, further supporting the concept that targeted manipulation of host regulatory components can improve plant defense responses (Yuan et al., 2026). Importantly, the enhanced resistance-associated phenotype reported here was linked to a CRISPR/Cas9-induced missense substitution. The T→G substitution observed at the target site is consistent with a repair outcome of CRISPR/Cas9-induced double-strand breaks through non-homologous end joining (NHEJ). Although insertions and deletions are the predominant outcomes of Cas9-mediated editing, localized nucleotide substitutions have also been reported following NHEJ in plants and other eukaryotic systems. This observation is biologically plausible because ion channels and regulatory proteins are often highly sensitive to single amino acid substitutions that may alter channel gating, protein conformation, or interactions with signaling partners. The leucine-to-glutamine substitution identified in the edited *StDND2* protein occurs within a conserved region and may subtly modify protein function without abolishing it entirely. This partial alteration of *StDND2* activity may contribute to the observed reduction in disease severity under the tested experimental conditions. Structural predictions generated using computational modeling suggested localized conformational changes associated with this substitution (**Figure 9**). However, these predictions provide qualitative insights only, and the functional impact on channel activity and calcium signaling remains hypothetical and will require experimental validation through biochemical or electrophysiological approaches. Direct measurement of calcium flux and downstream defense signaling responses will be necessary to confirm the functional consequences of the identified mutation.

In contrast to approaches that rely on strong constitutive defense activation, which can result in growth penalties, *StDND2* editing in the present study was associated with reduced disease severity without obvious visible developmental abnormalities under the tested conditions. Previous studies have demonstrated that CRISPR/Cas9-mediated modification of potato susceptibility genes, including *StDND1*, *StDMR6, StBIK1, StCeSA3*, and *StCHL1*, can reduce disease severity under control conditions (Gao et al., 2024; Karlsson et al., 2024; Manzoor et al., 2024; Mclaughlin et al., 2025; Zhao et al., 2025; Yuan et al., 2026). Together, these findings suggest that susceptibility gene editing may complement other resistance strategies for improving disease resistance in crop plants by modifying host factors required for pathogen success, potentially reducing selective pressure on pathogen populations (Kieu et al., 2021; Bi et al., 2023; Karlsson et al., 2024; Poudel et al., 2025). However, a common limitation associated with some S-gene knockouts is the occurrence of pleiotropic effects, including altered plant architecture, reduced vigor, or compromised yield. In contrast, the *StDND2* editing strategy described in this study was associated with reduced disease severity under controlled conditions without obvious visible developmental abnormalities under the tested conditions. This observation is particularly relevant for potato improvement studies, where maintenance of plant growth characteristics remains an important consideration (Xu et al., 2023; Neres and Wright, 2024; Liu et al., 2025; Van Butselaar et al., 2025). Compared with previous reports focusing on complete loss-of-function alleles, the nucleotide substitution identified in *StDND2* represents a comparatively moderate form of genome modification. Rather than causing complete disruption of gene activity, this alteration may partially modulate immune regulation, thereby contributing to reduced disease severity under the tested conditions without obvious visible developmental abnormalities. This observation is consistent with emerging evidence suggesting that partial modulation of susceptibility-associated factors may reduce the likelihood of severe fitness-related effects compared with complete gene disruption. Taken together, these findings highlight the potential value of targeting regulatory susceptibility genes such as *StDND2* for investigating disease resistance improvement in potatoes. Future studies may further evaluate the integration of susceptibility gene editing with complementary approaches such as immune receptor engineering and signaling pathway modulation under diverse experimental conditions.

Although *StDND2* editing was associated with reduced disease severity under controlled conditions, resistance was evaluated using detached leaf assays with a single *P. infestans* isolate, which may not fully reflect field performance. Therefore, the observed phenotype should be interpreted as reduced disease severity under experimental conditions rather than definitive field-level resistance. Validation under diverse environmental conditions and against multiple pathogen isolates will be necessary to further assess the breadth and stability of the observed response. Assessment of vegetative growth and morphology in the present study was primarily based on qualitative visual observations and tuber number measurements; comprehensive quantitative evaluation of growth-related traits such as plant height, biomass, canopy architecture, and leaf development was beyond the scope of this work and will require further investigation. Furthermore, analyses were limited to a subset of regenerated plants, and evaluation across additional independently regenerated lines and genetic backgrounds will be necessary to confirm the robustness and general applicability of the observed phenotype. The independence of editing events could not be conclusively confirmed based on the available data. In addition, the present study was conducted using T0 and T1 generation plants, and stable T2 homozygous lines were not evaluated; therefore, long-term stability and inheritance of the edited phenotype remain to be determined.

Although the observed phenotype is consistent with the proposed role of *DND2* as a negative regulator of immunity, the underlying molecular mechanisms remain to be elucidated. Detailed mechanistic characterization, including gene expression profiling, pathogen-induced regulation, subcellular localization, and analysis of calcium-mediated immune signaling pathways, was beyond the scope of this study and will be required to further understand the function of *StDND2* in potato. Importantly, the edited plants analyzed in this study did not exhibit obvious qualitative developmental abnormalities or reductions in tuber number under the tested conditions, although comprehensive quantitative phenotypic characterization was not performed. Overall, these findings provide preliminary evidence that targeted editing of *StDND2* is associated with reduced disease severity under controlled experimental conditions and supports further evaluation in advanced generations and field environments.

## Data Availability

The original contributions presented in the study are included in the article/**Supplementary Material**. Further inquiries can be directed to the corresponding author.
