# Supplementary material for: Genome editing of susceptibility gene StDND2 enhances Phytophthora resistance in Solanum tuberosum
Source: Front Plant Sci. 2026 Jun 5;17:1807632. doi: 10.3389/fpls.2026.1807632 (PMC13279601; doi:10.3389/fpls.2026.1807632)
Supplement: Supplementary file 1 [file Table1.docx]

**Supplementary Materials**

**Supplementary Methods**

**Vector Details**

The Ptz-p-chimera vector (Hameed et al., 2017) comprises the *Arabidopsis thaliana* U6-26 promoter, gRNA scaffold (sgRNA cassette), and kanamycin (Thermo Fisher Scientific, USA) as the selectable marker. A schematic representation of the Ptz-p-chimera vector architecture, including the U6 promoter and sgRNA scaffold, is shown in Supplementary Figure S1a. This vector facilitated cloning of the sgRNA cassette into the expression vector PK2-GW7-Cas9 (Ali et al., 2015). A *BbsI* site (New England Biolabs, USA) between the U6-26 promoter and gRNA scaffold enables target sequence insertion (Supplementary Figure S1a, b). The overall cloning workflow, including amplification, TA cloning, and final assembly into PK2-GW7-Cas9, is illustrated in Supplementary Figure S1c-e.


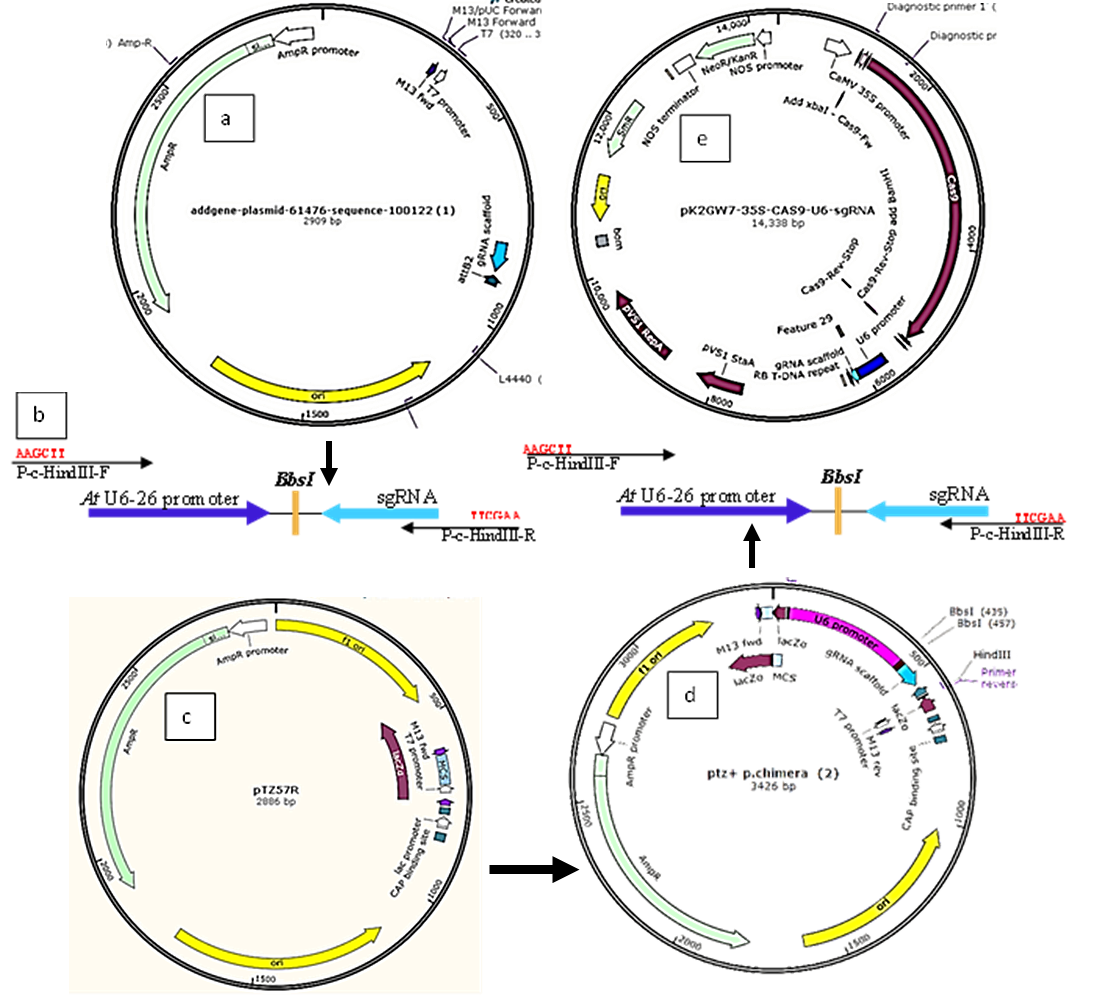


**Figure S1. Schematic representation of the cloning strategy used to assemble the U6-sgRNA cassette and its integration into the CRISPR/Cas9 expression vector PK2-GW7-Cas9.** (a) Plasmid map of the P-chimera cloning vector containing the *Arabidopsis thaliana* U6-26 promoter and gRNA scaffold (sgRNA cassette). (b) PCR amplification of the U6-26 promoter and sgRNA scaffold using primers containing *HindIII* restriction sites; a *BbsI* restriction site is located between the U6 promoter and sgRNA scaffold to facilitate target sequence insertion. (c) Plasmid map of the TA cloning vector Ptz57rt used for intermediate ligation of the amplified sgRNA cassette. (d) Plasmid map of the intermediate construct Ptz-p-chimera (Ptz57rt-U6-sgRNA cassette). (e) Plasmid map of the final CRISPR/Cas9 binary vector PK2-GW7-Cas9 containing the ligated U6-sgRNA cassette and the *StDND2* target sequence within the T-DNA region.

5


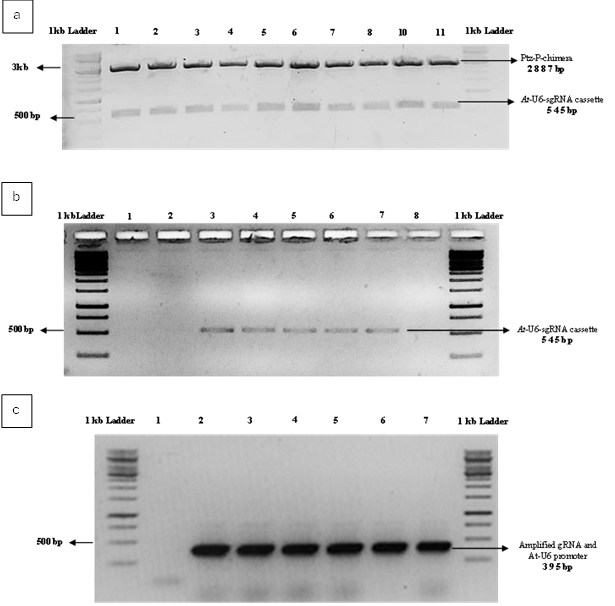


**Figure S2. Molecular validation of sgRNA cassette cloning and assembly.** (a) Agarose gel electrophoresis showing *HindIII* restriction digestion of the Ptz-p-chimera plasmid. The ~3 kb band corresponds to the vector backbone, while the ~545 bp band represents the released At U6–sgRNA cassette. (b) PCR amplification of the At U6–sgRNA cassette from the Ptz-p-chimera vector, showing the expected ~545 bp product. Lanes 1–2 represent no-template controls, while lanes 3–8 show successful amplification of the sgRNA cassette. (c) PCR confirmation of sgRNA cassette insertion into the PK2-GW7-Cas9 vector using primer p-c-*HindIII*-F and the corresponding *DND2*-gRNA reverse primer. Lane 1 represents the negative control, and lanes 2–7 show the expected ~395 bp amplicon confirming successful ligation.

**Next-Generation Sequencing and Data Analysis**

Amplicons spanning the CRISPR/Cas9 target site in *StDND2* were sequenced using Illumina-based next-generation sequencing platforms across two generations (T0 and T1). For the T0 generation, sequencing libraries were prepared and run in two independent pools: **210527_JM5G8** (10 samples) and **220211_K7GDR** (5 samples). The initial pool (210527_JM5G8) showed evidence of contamination and was therefore excluded from downstream analyses; consequently, mutation analysis was conducted using data from the second T0 pool (220211_K7GDR). For the T1 generation, **five independent samples** were sequenced to confirm inheritance and stability of the edited allele. Raw sequencing reads were subjected to quality control (QC) analysis using **FastQC v0.12.1,** which assessed per-base sequence quality, GC content, sequence duplication levels, overrepresented sequences, and adapter contamination. Individual FastQC reports were aggregated using **MultiQC v1.17** to generate a consolidated QC summary. Low-quality bases and residual adapter sequences were removed using **fastp v0.20.1** (Chen et al., 2018). Post-filtering read quality was reassessed using FastQC to ensure that only high-quality reads were retained for downstream analyses. Filtered reads were aligned to the StDND2 reference sequence using the **Burrows–Wheeler Aligner (BWA) v0.7.17-r1188,** with reference indices generated prior to alignment (Barrett et al., 2013) Read alignment was performed using the **bwa-mem** algorithm. Resulting SAM files were converted to BAM format, sorted, and indexed using **SAMtools v1.13** (Danecek et al., 2021). Alignments were visualized using the **Integrative Genomics Viewer (IGV)** to confirm coverage and mutation patterns at the target locus. To eliminate PCR-derived artifacts, duplicate reads were removed using the **MarkDuplicates** function of **PICARD v2.25.4.** Deduplicated BAM files were subsequently subjected to variant calling using the **Haplotype Caller** tool of the **Genome Analysis Toolkit (GATK) v4.2.1.0,** which identifies single nucleotide polymorphisms (SNPs) based on mismatches between aligned reads and the reference sequence. Identified variants corresponding to CRISPR/Cas9-induced edits at the StDND2 locus are shown in **Figure 5.**


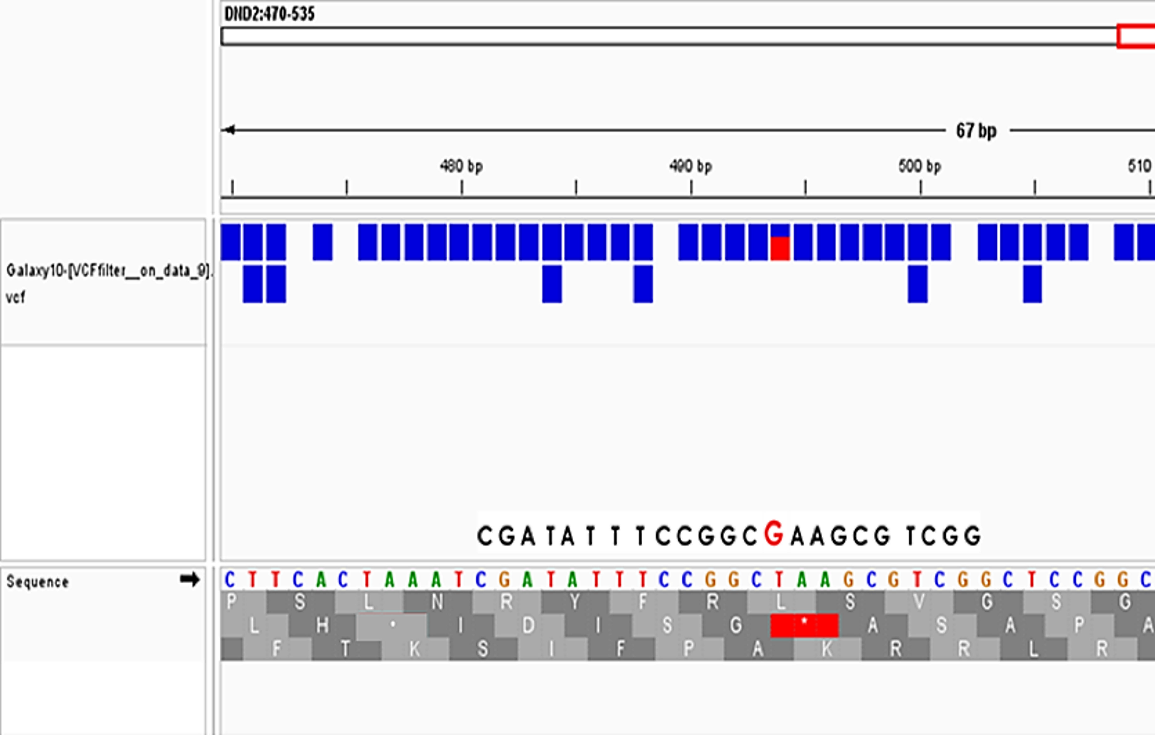


**Figure S3: IGV-based visualization of CRISPR/Cas9-induced mutation in *StDND2***

Integrative Genomics Viewer (IGV) screenshot showing aligned sequencing reads at the *StDND2* target locus. The consistent T→G substitution at the predicted Cas9 cleavage site is highlighted in red within the aligned reads. The reference sequence is shown below, with the corresponding amino acid translation displayed in the lower panel. The uniform presence of the edited nucleotide across aligned reads indicates a consistent editing pattern at the target site. *The absence of wild-type reads at this position supports a consistent editing signal across allelic copies in the analyzed lines.*


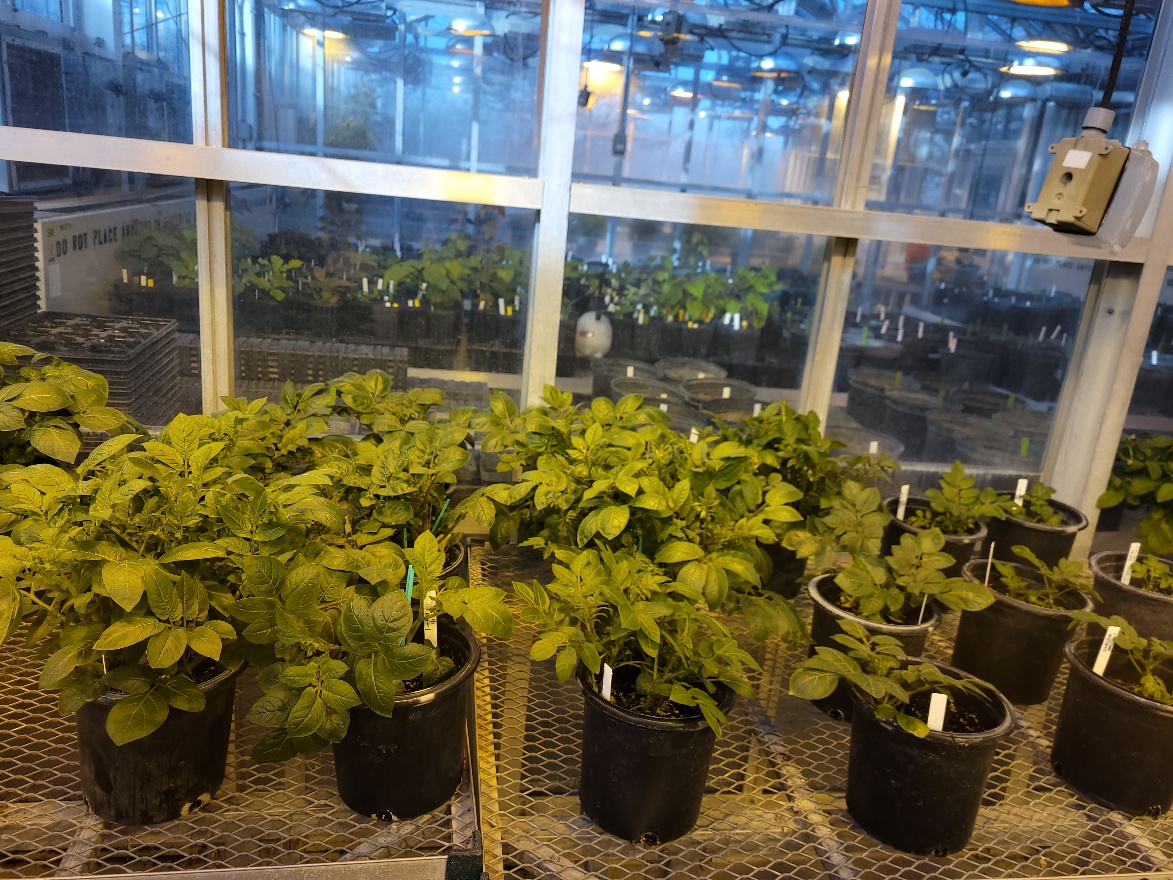


**Figure S4. Representative vegetative growth of *StDND2*-edited and empty-vector control potato plants under controlled greenhouse conditions.**

Plants were grown under same environmental conditions, and no visible differences in overall morphology, growth habit, or leaf development were observed between edited lines and control plants.

**Wild type *StDND2* Sequence**

KNERREGENESGDRDSPRERNSNSLL.GTIKSNYIYSIFELIVCYYIQFSLFITFK.LYLMCNFGIYFTFDELINVRKSPYYHLFLIYTSSQKNKLKSNFLHLNDNIHSPKFVYGLKLKVIIGIPPWLPPTTASDSSPTGNSRGFAPSLNRYFR**LSVGSGS**ILLTISTFHVSHRSTLYYFIALSRVRRNCYCLNYSYEEKFGLKFCIFVKNIHFICTKNLFRTYKLEIYLVGVMIFVKQNGFPV

**Mutated *StDND2* Sequence**

KNERREGENESGDRDSPRERNSNSLLGTIKSNYIYSIFELIVCYYIQFSLFITFKLYLMCNFGIYFTFDELINVRKSPYYHLFLIYTSSQKNKLKSNFLHLNDNIHSPKFVYGLKLKVIIGIPPWLPPTTASDSSPTGNSRGFAPSLNRYFR**QSVGSGS**ILLTISTFHVSHRSTLYYFIALSRVRRNCYCLNYSYEEKFGLKFCIFVKNIHFICTKNLFRTYKLEIYLVGVMIFVKQNGFPV

Table S1. **Reciprocal** **Protein BLAST for AtDND2 for StDND2 Orthology Confirmation**

| **Top hit gene accession** | **Alignment length** | **Percent ID** | **Specie** |
| --- | --- | --- | --- |
| PGSC0003DMG400025027 | 349 | 78.2 | Solanum tuberosum |
| Solyc12g005400.2 | 349 | 78.8 | Solanum lycopersicum |
| T459_33278 | 349 | 79.1 | Capsicum annum |

**Table S2. Reverse BLAST of Solanum tuberosum top hit for Arabidopsis DND2 against different species**

| **Top hit gene accession** | **Alignment length** | **Percent ID** | **Specie** | **Function** |
| --- | --- | --- | --- | --- |
| Solyc10g006800.3 | 417 | 94 | Solanum lycopersicum | **Cyclic nucleotide-binding domain-containing protein** |
| T459_33278 | 404 | 91.1 | Capsicum annum | Cyclic nucleotide-gated ion channel 4 |
| CNGC4 AT5G54250 | 403 | 75.4 | Arabidopsis | cyclic nucleotide-gated cation channel 4 |


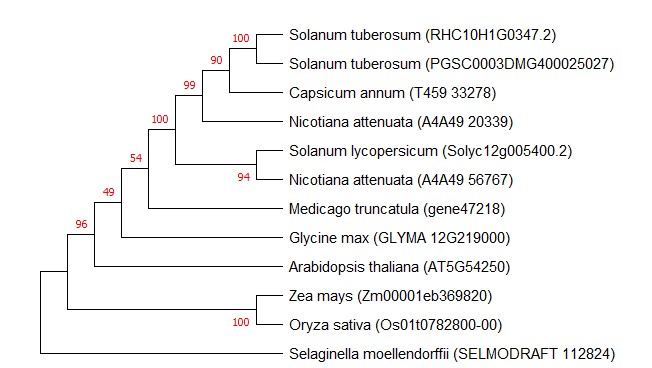


**Figure S5. Evolutionary analysis by Maximum Likelihood method**

The evolutionary history was inferred by using the Maximum Likelihood method and JTT matrix-based model [1]. The bootstrap consensus tree inferred from 1000 replicates [3] is taken to represent the evolutionary history of the taxa analyzed. Branches corresponding to partitions reproduced in less than 50% bootstrap replicates are collapsed. The percentage of replicate trees in which the associated taxa clustered together in the bootstrap test 1000 replicates) are shown next to the branches [3]. Initial tree(s) for the heuristic search were obtained automatically by applying Neighbor-Join and BioNJ algorithms to a matrix of pairwise distances estimated using the JTT model, and then selecting the topology with superior log likelihood value. This analysis involved 12 amino acid sequences. The sequence of Selaginella moelledorffii was taken as outgroup. All positions with less than 95% site coverage were eliminated, i.e., fewer than 5% alignment gaps, missing data, and ambiguous bases were allowed at any position (partial deletion option). There were a total of 432 positions in the final dataset. Evolutionary analyses were conducted in MEGA11 [2]

**References**

Ali, Z., Abul-Faraj, A., Li, L., Ghosh, N., Piatek, M., Mahjoub, A., Aouida, M., Piatek, A., Baltes, Nicholas j., Voytas, Daniel f., Dinesh-Kumar, S., and Mahfouz, Magdy m. (2015). Efficient Virus-Mediated Genome Editing in Plants Using the CRISPR/Cas9 System. *Molecular Plant* 8**,** 1288-1291.

Barrett, T., Wilhite, S.E., Ledoux, P., Evangelista, C., Kim, I.F., Tomashevsky, M., Marshall, K.A., Phillippy, K.H., Sherman, P.M., Holko, M., Yefanov, A., Lee, H., Zhang, N., Robertson, C.L., Serova, N., Davis, S., and Soboleva, A. (2013). NCBI GEO: archive for functional genomics data sets--update. *Nucleic Acids Res* 41**,** D991-995.

Chen, S., Zhou, Y., Chen, Y., and Gu, J. (2018). fastp: an ultra-fast all-in-one FASTQ preprocessor. *Bioinformatics* 34**,** i884-i890.

Danecek, P., Bonfield, J.K., Liddle, J., Marshall, J., Ohan, V., Pollard, M.O., Whitwham, A., Keane, T., Mccarthy, S.A., Davies, R.M., and Li, H. (2021). Twelve years of SAMtools and BCFtools. *Gigascience* 10.

Hameed, A., Tahir, M.N., Asad, S., Bilal, R., Van Eck, J., Jander, G., and Mansoor, S. (2017). RNAi-mediated simultaneous resistance against three RNA viruses in potato. *Molecular biotechnology* 59**,** 73-83.

Jones D.T., Taylor W.R., and Thornton J.M. (**1992**). The rapid generation of mutation data matrices from protein sequences. *Computer Applications in the Biosciences***8**: 275-282.

Tamura K., Stecher G., and Kumar S. (**2021**). MEGA 11: Molecular Evolutionary Genetics Analysis Version 11. *Molecular Biology and Evolution* https://doi.org/10.1093/molbev/msab120.

Felsenstein J. (**1985**). Confidence limits on phylogenies: An approach using the bootstrap. *Evolution* **39**:783-791.
